# Supplementary material for: Neuropilin 1 regulates bone marrow vascular regeneration and hematopoietic reconstitution
Source: Nat Commun. 2021 Nov 30;12:6990. doi: 10.1038/s41467-021-27263-y (PMC8635308; doi:10.1038/s41467-021-27263-y)
Supplement: Supplementary file 1 — Supplementary Information [file 41467_2021_27263_MOESM1_ESM.pdf]

Supplementary Figure 1

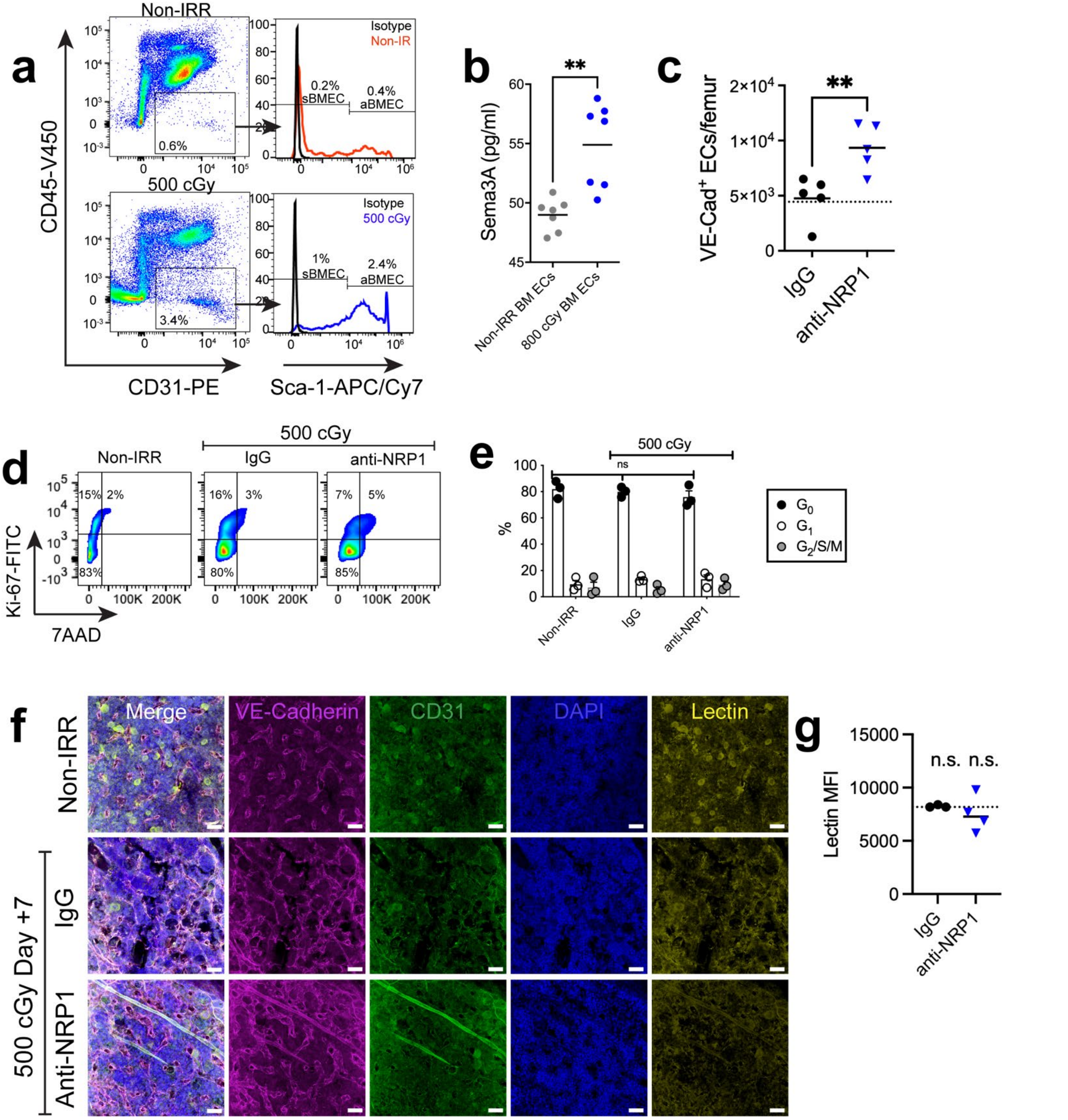

Supplementary Figure 1. NRP1 inhibition promotes BM EC regeneration. a Representative gating used to isolate CD31$^+$Sca-1$^+$ aBMECs and CD31$^+$Sca-1$^-$ sBMECs for qRT-PCR analyses. **b** SEMA3A protein levels by ELISA in the culture supernatant of mouse BM ECs at baseline and at 24 hours after 800 cGy irradiation (n = 7 replicates/condition; p=0.0015). **c** Numbers of BM ECs in mice at day +7

following 500 cGy TBI and treatment with IgG or anti-NRP1 (n = 5/group; p=0.0046). Dotted line represents numbers in non-irradiated mice. **d** Representative flow cytometric analysis and (**e**) bar graphs showing the percentages of $CD45^{-}VE\text{-}cad^{+}$ BM ECs in G_0, G_1 or G_2/S/M phase, based on Ki67/7AAD analysis, in *C57BL/6* mice at day +5 following 500 cGy TBI and the treatments shown (n=3/group, data are presented as mean values +/- SEM; Non-IRR vs IgG: p=0.8662; Non-IRR vs Anti-NRP1: p=0.4016). **f** Microscopic images of femur sections stained for VE-Cadherin (magenta), CD31 (green), Lectin (yellow) and nuclei (DAPI) and (**g**) quantification of lectin MFI. Dotted line shows non-IRR control levels (n-3=5 fields of view; Non-IRR vs IgG: p=0.4252; Non-IRR vs Anti-NRP1: p=0.5533). Data were assessed by two-tailed student's unpaired t-test (**b**) and (**c**), two-way ANOVA followed by Holm-Sidak's corrected two-sided t-test (**e**) for comparisons across each cell cycle phase, and one-sample two-sided t-test and Wilcoxon test (**g**) compared to non-IRR control. **p < 0.01. Source data are provided as a Source Data file.

Supplementary Figure 2

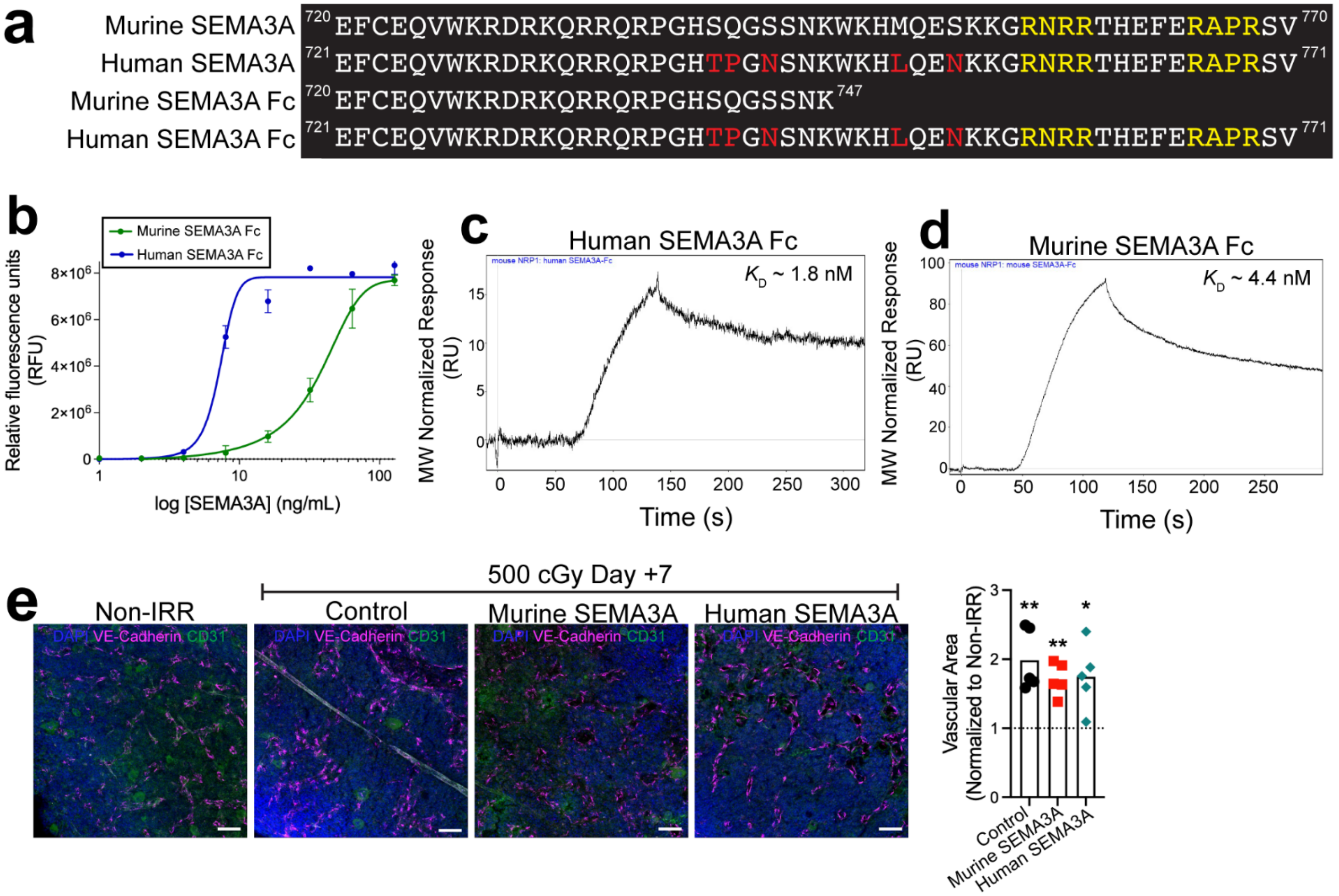

Supplemental Figure 2. Effects of administration of high binding affinity SEMA3A on BM vascular recovery from TBI. a Sequence alignment of full-length native mouse SEMA3A (Glu720-Val770) and human SEMA3A (Glu721-Val771) amino acid sequence compared to commercially available truncated, mouse SEMA3A Fc (Glu720-Lys747) and human SEMA3A Fc (Glu721-Val771) recombinant proteins. Red marks sequence differences between mouse and human SEMA3A amino acid residues. Yellow shows the conserved RNRR and RAPR high affinity binding domains. **b** Sandwich NRP1 ELISA to determine the binding capacity of human SEMA3A and truncated murine SEMA3A to the N-terminal end of mouse NRP1 (n=4 replicates/condition, data are presented as mean values +/- SEM). **c** Surface plasmon resonance (SPR) analysis of the binding of human SEMA3A Fc to murine NRP1 is shown, along with equilibrium dissociation constant (K_D). **d** SPR analysis of binding of truncated murine SEMA3A to murine NRP1, with associated K_D. **e** Microscopic analysis and quantification of the BM vascular area at day +7 of mice irradiated (500 cGy) and treated with human

SEMA3A Fc or murine SEMA3A Fc every other day. Scale bar = 20μm. (n=4-5 replicates/condition; Control: $p=0.0079$; mouse SEMA3A: $p=0.0026$; human SEMA3A: $p=0.0245$). Statistics denote one-sample two-sided t-test and Wilcoxon test compared to non-IRR control vascular area. *$p < 0.05$, **$p < 0.01$. Source data are provided as a Source Data file.

Supplementary Figure 3

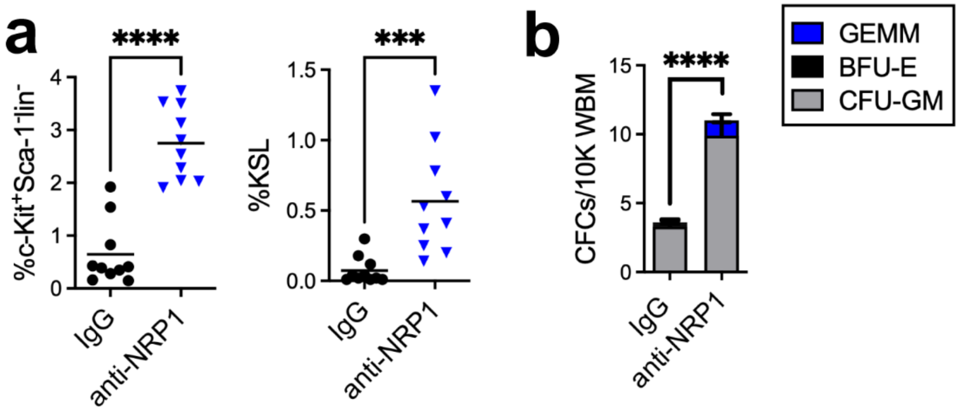

Supplementary Figure 3. NRP1 inhibition promotes hematopoietic regeneration. a Percentages of BM c-kit$^+$sca1$^-$lin$^-$ cells and KSL cells per femur at day +10 following 500 cGy TBI and treatment with IgG or anti-NRP1 (n = 5-10 mice/condition; %c-Kit+Sca-1-lin-: p<.0001; %KSL: p<.0006). **b** Numbers of BM CFCs in mice at day +10 post-500 cGy TBI and treatments shown (n = 5 mice/group, data are presented as mean values +/- SEM; p<0.0001). Data were analyzed using a student's unpaired two-sided t-test (**a**) or a two-way ANOVA followed by a Holm-Sidak's multiple comparison two-sided t-test (**b**). ***p < 0.001, ****p<0.0001. Source data are provided as a Source Data file.

Supplementary Figure 4

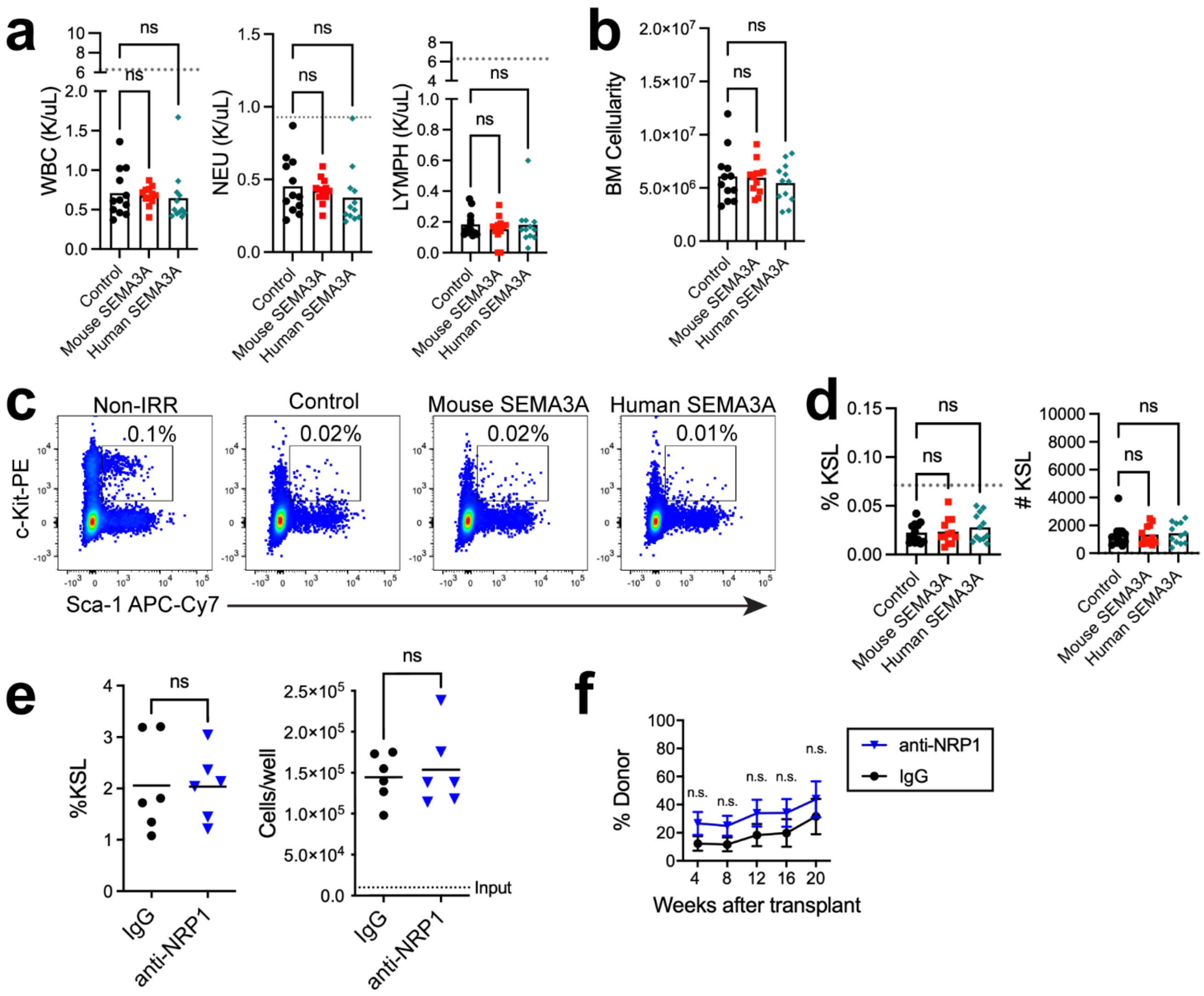

Supplementary Figure 4. Effects of administration of high binding affinity SEMA3A on hematopoietic recovery following TBI. a Complete blood count analysis and (**b**) BM cellularity at day +10 in mice irradiated with 500 cGy TBI and treated with 2 µg recombinant human SEMA3A Fc, or murine SEMA3A Fc, every other day (n=2 independent experiments with n=5-7 mice per treatment group/experiment; mean is shown and statistics denote Holm-Sídák's multiple comparisons test after one-way ANOVA; WBC: Mouse SEMA3A: p=0.9828; Human SEMA3A: p=0.9828; NEU: Mouse SEMA3A: p=0.6727; Human SEMA3A: p=0.5667; LYMPH: Mouse SEMA3A: p=0.9984; Human SEMA3A: p=0.9984). **c** Representative flow cytometric analysis and (**d**) quantification of the percentages and numbers of BM KSL cells in mice at day +10 after irradiation (500 cGy) and treatment

with human SEMA3A Fc or murine SEMA3A Fc every other day. (n=2 independent experiments with n=5-7 mice/experiment; %KSL: Mouse SEMA3A: p=0.8566; Human SEMA3A: p=0.6209; #KSL: Mouse SEMA3A: p=0.989; Human SEMA3A: p=0.9387). **e** At left, percentages of KSL cells at 1 week of culture following irradiation with 300 cGy and treatment with complete media supplemented with 10 ug/ml IgG or 10 ug/ml anti-NRP1. At right, numbers of KSL cells at 1 week of culture in the treatment conditions shown (n=6 replicates/condition; %KSL: p=0.965; Cells/well: p=0.6671). **f** PB donor $CD45.2^+$ cell engraftment over time in $CD45.1^+$ *B6.SJL* mice transplanted with 3×10^6 BM cells collected at 20 weeks from primary recipient mice that were previously transplanted with BM cells from irradiated, anti-NRP1 treated donors or irradiated, IgG-treated donors, along with 2×10^5 competitor $CD45.1^+$ BM cells (n = 6-7 mice/condition, data are presented as mean values +/- SEM; p=0.75 for all timepoints). Data were assessed by one-way ANOVA followed by Holm-Sidak's multiple comparison two-sided t-tests (**a**, **b**, **d**), unpaired two-sided t-tests (**e**), and two-way ANOVA followed by Holm-Sidak's multiple comparison two-sided t-tests (**f**). **p < 0.01, ***p < 0.001, ****p < 0.0001. Source data are provided as a Source Data file.

Supplementary Figure 5

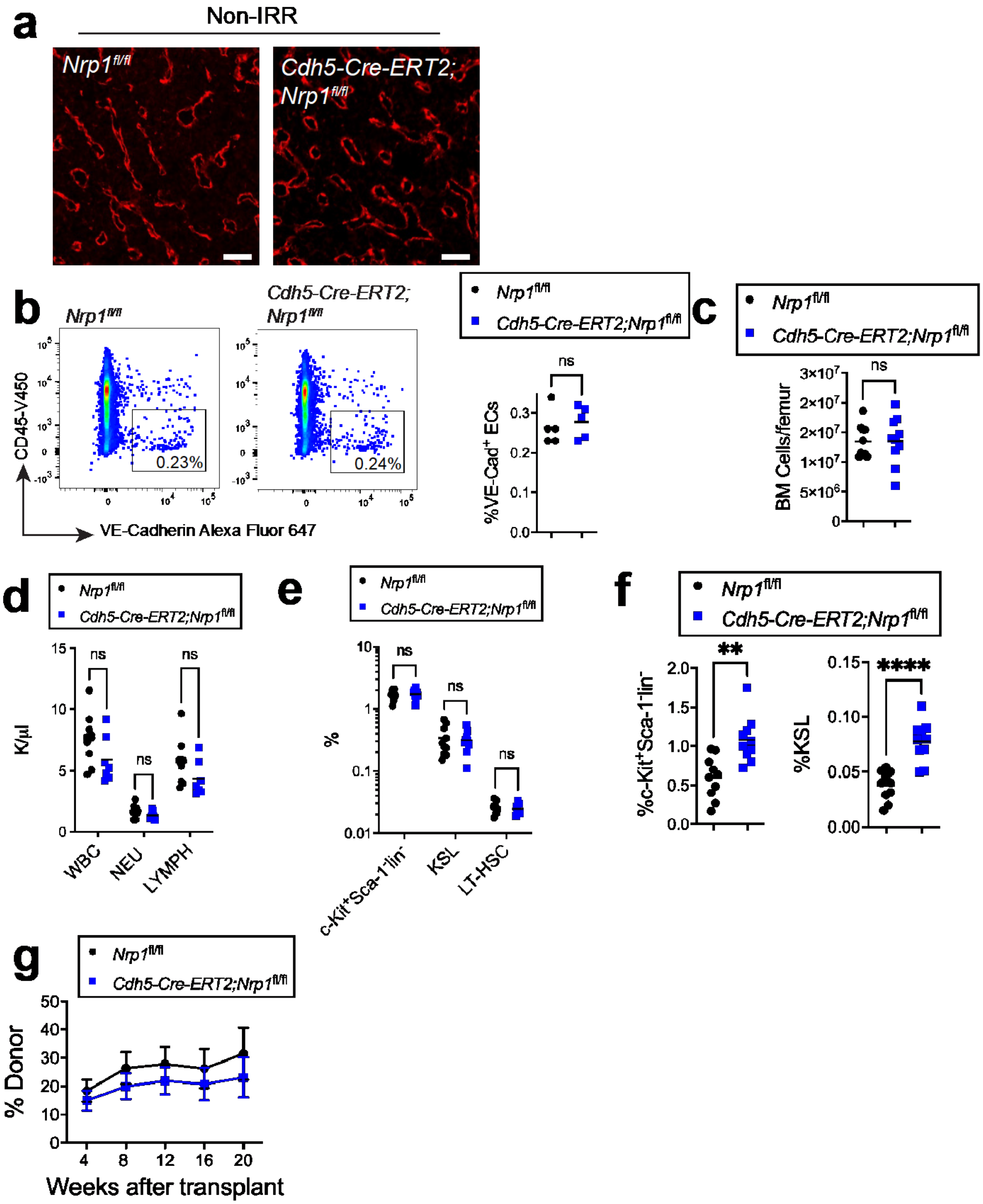

Supplementary Figure 5. Baseline effects of EC-specific *Nrp1* deletion. a Representative microscopic images of VE-Cad^{+} BM vessels (red) in femur sections from *Nrp1$^{fl/fl}$* mice and *Cdh5-Cre-ERT2;Nrp1$^{fl/fl}$* mice following tamoxifen treatment. Scale bar, 100 μm. **b** At left, representative flow cytometric analysis of CD45^{-}VE-Cad^{+} BM ECs from tamoxifen-treated *Nrp1$^{fl/fl}$* mice and *Cdh5-Cre-ERT2;Nrp1$^{fl/fl}$* mice. At right, mean percentages of BM ECs in each group of mice (n = 5 mice/group; p=0.6207). **c** BM cell counts in tamoxifen-treated *Nrp1$^{fl/fl}$* mice and *Cdh5-Cre-ERT2;Nrp1$^{fl/fl}$* mice (n = 10 mice per group; p=0.9764). **d** PB WBC, NEU and LYMPHs in non-IRR *Nrp1$^{fl/fl}$* mice and *Cdh5-Cre-ERT2;Nrp1$^{fl/fl}$* mice following tamoxifen treatment (n = 7-10 mice/group; WBC: p=0.0743; NEU: p=0.6463; LYMPH: p=0.1377). **e** Baseline percentages of BM c-kit^{+}sca1^{-}lin^{-} cells, KSL stem/progenitor cells and CD150^{+}CD41^{-}CD48^{-} KSL cells (LT-HSCs) in the mice shown (n = 7-10 mice/group; p=0.9911). **f** Percentages of c-kit^{+}scal-1^{-}lin^{-} and KSL cells in the mice shown at day +10 following 500 cGy TBI (n = 10 mice/condition; %c-Kit^{+}Sca-1^{-}lin^{-}: p=0.0012; %KSL: p<0.0001). **g** PB donor CD45.2^{+} cell engraftment over time in CD45.1^{+} *B6.SJL* mice transplanted with 5 x 10^{5} BM cells collected at day +10 from 500 cGy – irradiated *Nrp1$^{fl/fl}$* mice and *Cdh5-Cre-ERT2;Nrp1$^{fl/fl}$* mice (CD45.2^{+}), along with 2 x 10^{5} CD45.1^{+} competitor BM cells (n = 10-11 mice/group, data are presented as mean values +/- SEM). Data were assessed by Student's two-tailed t test (**b**, **c**, **f**) and two-way ANOVA followed by Holm-Sidak's multiple comparison two-sided t-tests (**d**, **e**, **g**). **p < 0.01, ****p < 0.0001. Source data are provided as a Source Data file.

Supplementary Figure 6

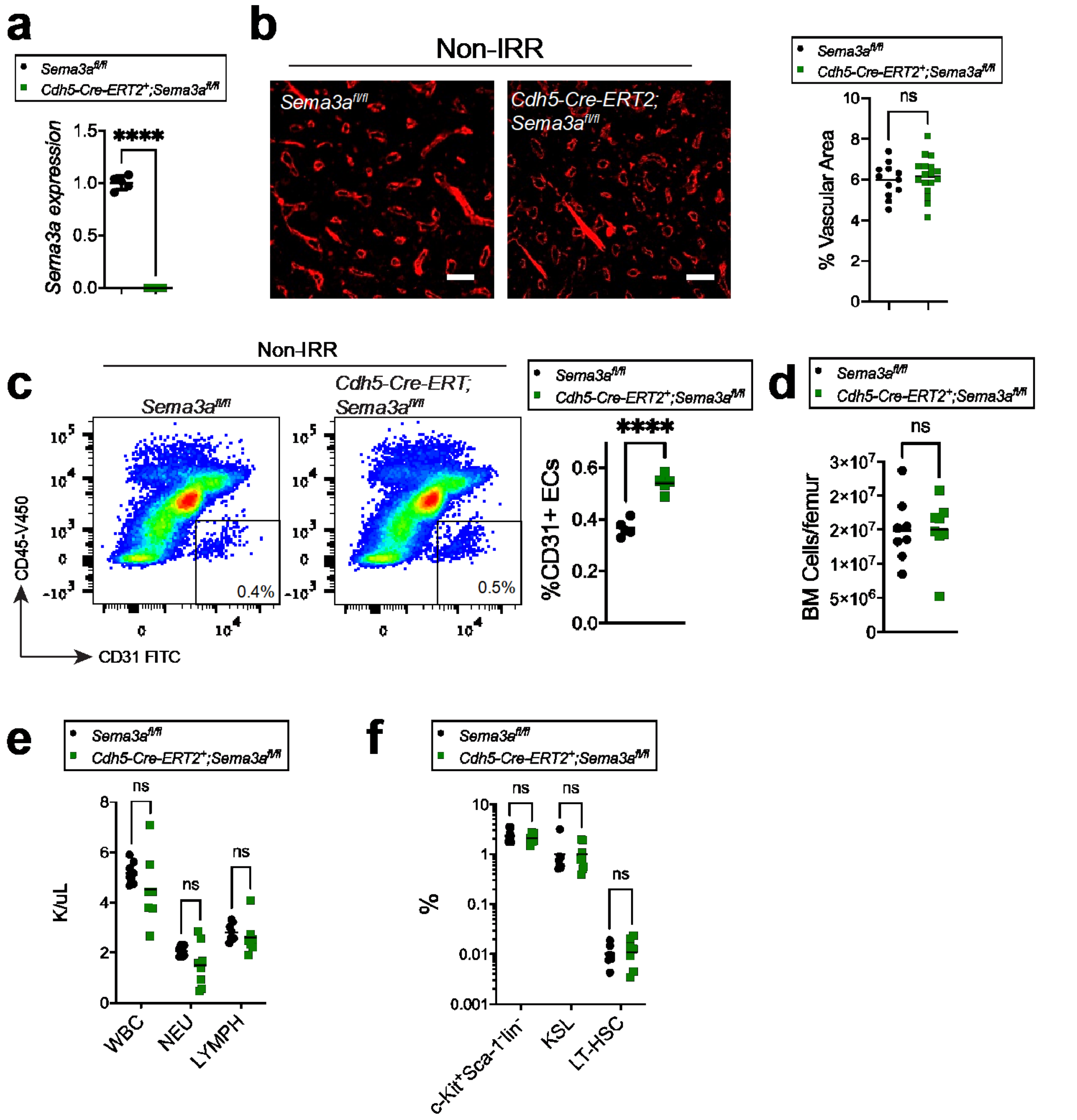

Supplementary Figure 6. Baseline effects of EC-specific deletion of *Sema3a*. a qRT-PCR analysis of *Sema3a* expression in VE-cad+ BM ECs from *Sema3a*$^{fl/fl}$ mice and *Cdh5-Cre-ERT2;Sema3a*$^{fl/fl}$ mice

following tamoxifen treatment (n = 5 mice/group, two-tailed Student's t test; p<0.0001). **b** At left, representative images of VE-cad$^+$ BM vessels (red) in femur sections from non-IRR *Sema3a*$^{fl/fl}$ mice and *Cdh5-Cre-ERT2;Sema3a*$^{fl/fl}$ mice following tamoxifen treatment. Scale bar, 100 μm. At right, quantification of BM vascular area per field (n = 12-17 replicates/group; p=0.5893). **c** At left, representative flow cytometric analysis of CD45$^-$CD31$^+$ BM ECs in non-IRR, tamoxifen-treated *Sema3a*$^{fl/fl}$ mice and *Cdh5-Cre-ERT2;Sema3a*$^{fl/fl}$ mice. At right, percentages of BM ECs within the mice groups shown (n = 5 mice/group, student's two-tailed t test; p<0.0001). **d** BM cell counts/femur in non-IRR, tamoxifen-treated *Sema3a*$^{fl/fl}$ mice and *Cdh5-Cre-ERT2;Sema3a*$^{fl/fl}$ mice (n = 8 mice/group; p=0.9688). **e** PB WBC, NEU and LYMPHs in non-IRR *Sema3a*$^{fl/fl}$ mice and *Cdh5-Cre-ERT2; Sema3a*$^{fl/fl}$ mice (n = 8 mice/group; WBC: p=0.2822; NEU: p=0.2822; LYMPH: p=0.613). **f** Percentages of BM c-kit$^+$sca1$^-$lin$^-$ cells, KSL cells and LT-HSCs at baseline in *Sema3a*$^{fl/fl}$ mice and *Cdh5-Cre-ERT2;Sema3a*$^{fl/fl}$ mice (n = 8 mice/group; c-kit$^+$sca1$^-$lin$^-$: p=0.7372; KSL: p=0.9996; LT-HSC: p=0.9996). Data were assessed by unpaired two-sided t-test (**a**, **b**, **c**, **d**) and two-way ANOVA with Holm-Sidak's unpaired two-sided t-test (**e**, **f**). ****p < 0.0001. Source data are provided as a Source Data file.

Supplementary Figure 7

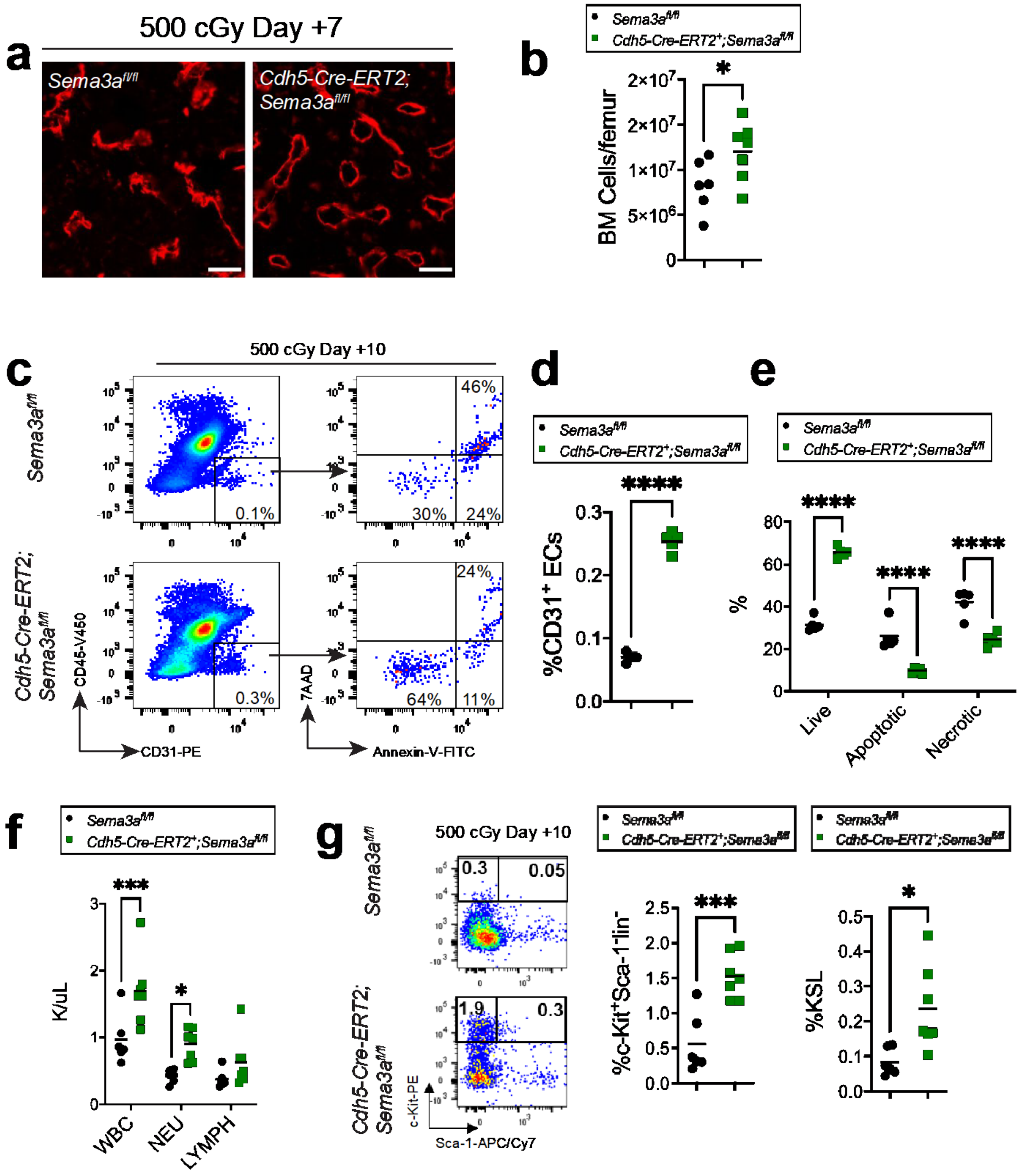

Supplementary Figure 7. EC – specific deletion of *Sema3a* accelerates BM vascular and hematopoietic regeneration. a Representative microscopic images of VE-cad$^+$ BM blood vessels (red) in femur sections from *Sema3a$^{fl/fl}$* mice and *Cdh5-Cre-ERT2;Sema3a$^{fl/fl}$* mice at day +7 following 500 cGy TBI. Scale bar, 50 μm. **b** BM cell counts in *Sema3a$^{fl/fl}$* mice and *Cdh5-Cre-ERT2;Sema3a$^{fl/fl}$*

mice at day +10 following 500 cGy TBI (n = 6-7 mice/group; p=0.0472). **c** At left, representative flow cytometric analysis of $CD45^-CD31^+$ BM ECs within BM lin^- cells from *Sema3a$^{fl/fl}$* mice and *Cdh5-Cre-ERT2;Sema3a$^{fl/fl}$* mice at day +10 following 500 cGy TBI. At right, percentages of $Annexin^+7AAD^-$ and $Annexin^+7AAD^+$ BM ECs are shown. **d** Mean percentages BM $CD31^+$ ECs at day +10 in the groups shown (n = 5 mice per group; p<0.0001). **e** Mean percentages of Live, Apoptotic and Necrotic BM ECs at day +10 following 500 cGy TBI in the groups shown (n = 5 mice/group; p<0.0001). **f** PB WBC, NEU and LYMPHs at day +10 following 500 cGy TBI in *Sema3a$^{fl/fl}$* mice and *Cdh5-Cre-ERT2;Sema3a$^{fl/fl}$* mice (n = 6-7 mice/group; WBC: p=0.0004; NEU: p=0.014). **g** At left, representative flow cytometric analysis of c-kit^+sca-$1^-$$lin^-$ progenitors and KSL stem/progenitor cells at day +10 following 500 cGy TBI. At right, mean percentages of c-kit^+sca-$1^-$$lin^-$ cells and KSL cells in the groups shown (n = 6-7 mice/group; c-kit^+sca-$1^-$$lin^-$: p=0.0006; KSL: p=0.0124). Data were assessed by two-tailed Student's t test (**b**, **d**, **g**) and two-way ANOVA with Sidak's multiple comparison test (**e**, **f**). *p < 0.05, **p < 0.01, ***p < 0.001. Source data are provided as a Source Data file.

Supplementary Figure 8

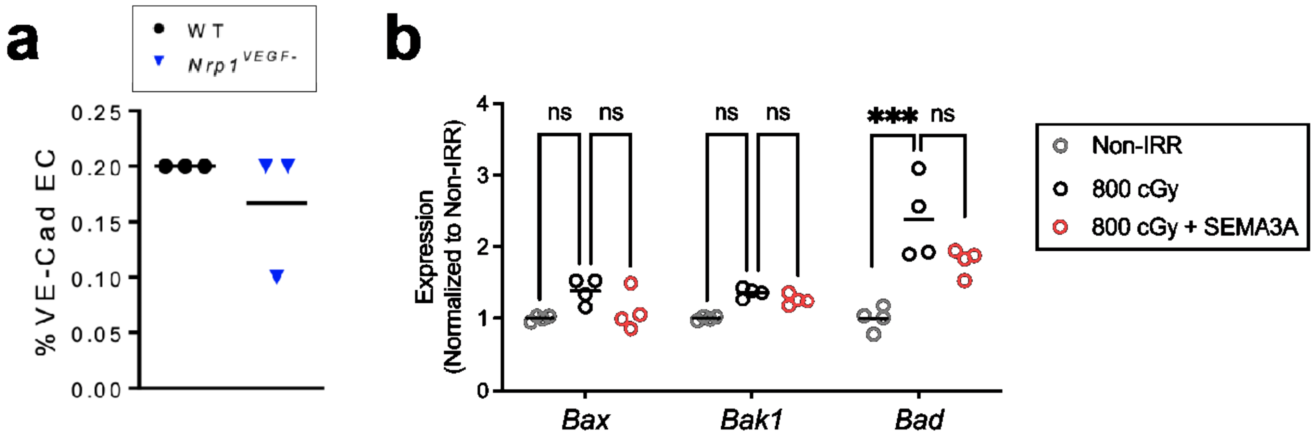

Supplementary Figure 8. Effects of irradiation and SEMA3A on apoptosis regulatory genes. **a** Percentages of VE-cad^{+} BM ECs at baseline in *Nrp1^{VEGF-}* mice and littermate controls (WT) (n = 3 mice/group). **b** qRT-PCR analysis of *Bax, Bak1, and Bad* expression in murine BM ECs at 6 hours following 800 cGy irradiation and treatment with or without 100 ng/ml SEMA3A. Data are normalized to non-IRR samples (n = 5 replicates/condition; *Bax*: Non-IRR vs 800 cGy: p=0.0557; Non-IRR vs 800 cGy + SEMA3A: p=0.0938; *Bak1*: Non-IRR vs 800 cGy: p=0.0769, Non-IRR vs 800 cGy + SEMA3A: p=0.5489; *Bad*: Non-IRR vs 800 cGy: p<0.0001; Non-IRR vs 800 cGy + SEMA3A: p=0.0021). Data were assessed by two-way ANOVA with Holm-Sidak's multiple comparison test (**b**). ***p<0.001. Source data are provided as a Source Data file.

Supplementary Figure 9

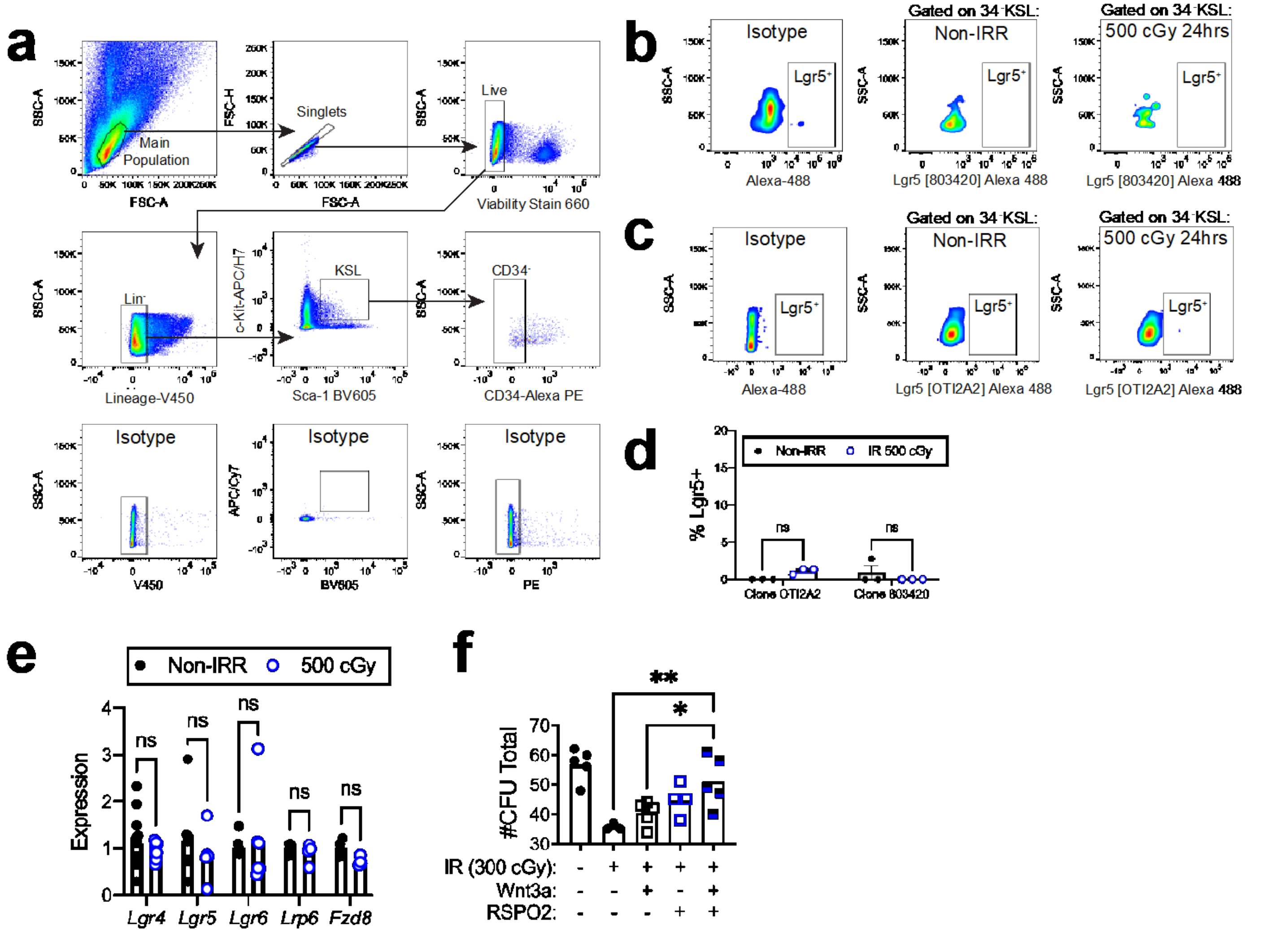

Supplementary Figure 9. Analysis of R spondin 2 receptor expression in BM HSCs following TBI. **a** Sorting strategy used to analyze KSL CD34^{-}KSL HSCs from n=5 *C57BL/6* mice for subsequent protein and RNA expression analyses. **b** Pseudocolor plot showing the expression of LGR5 on CD34^{-} KSL cells harvested from non-IRR *C57BL/6* mice and at 24 hours following 500 cGy TBI using antibody clone 803420, (**c**) clone OTI2A2, and (**d**) corresponding quantification of expression (n = 3 replicates/clone/condition, data are presented as mean values +/- SEM; p=0.2531). **e** qRT-PCR analysis for the expression of *Lgr4, Lgr5, Lgr6, Lrp6,* and *Fzd8* expression in BM CD34^{-}KSL cells isolated from *C57BL/6* mice following 500 cGy TBI (for non-IRR: *Lgr4,* n=15 replicates, *Lgr5*, n=10 replicates, *Lgr6,* n=8 replicates, *Lrp6*, n=5 replicates, *Fzd8*, n=4 replicates; for IRR: *Lgr4,* n=9 replicates,

Lgr5, n=*5* replicates, *Lgr6,* n=6 replicates, *Lrp6* and Fzd8 n=4 replicates; Comparisons, *Lgr4*: p=0.5594; *Lgr5*: p=0.3025; *Lgr6*: p=0.5913; *Lrp6*: p=0.7633; *Fzd8*: p=0.4244). **f** Total numbers of CFCs are shown at 48 hours following 300 cGy irradiation of BM ckit^{+}lin^{-} progenitor cells and culture with complete media supplemented with and without Wnt3a alone, R spondin 2 alone or R spondin 2 + Wnt3a (n = 3-5/group; IRR vs RSPO2 + WNT3A + IRR, p=.0077; IRR vs IRR + RSPO2: p=0.0283). Data were assessed by two-way ANOVA with Holm-Sidak's multiple comparison test (**d**, **e**) and one-way ANOVA with Holm-Sidak's multiple comparison test (**f**). *p < 0.05, **p < 0.01. Source data are provided as a Source Data file.

Supplementary Table 1

REAGENT or RESOURCE	SOURCE	IDENTIFIER
Antibodies		
Goat anti-Nrp1 (1:200)	R&D Systems	Cat. AF566; RRID:AB_355445
Anti-mouse VE-Cadherin Alexa Fluor® 647 (1:200)	Biolegend	Cat. 138006 RRID:AB_10569114
Anti-Leptin receptor PE (1:200)	Bioss Antibodies	Cat. bs-0961R-PE RRID:AB_11051477
Rat Anti-Mouse B220 (CD45R) APC-Cy7 (2µl/10^6 cells)	BD Biosciences	Cat.552094 RRID:AB_394335
Rat Anti-Mouse CD45 V450 (2µl/10^6 cells)	BD Biosciences	Cat.560501; RRID:AB_1645275
Rabbit Anti-P53 (2µl/10^6 cells)	Cell Signaling	Cat: 2527S RRID:AB_10695803
Mouse Anti-Phospho P53 (S15) (2µl/10^6 cells)	Cell Signaling	Cat: 9286S RRID:AB_331741
Mouse Anti-CD45.2 FITC Mouse anti-Mouse (2µl/10^6 cells)	BD Biosciences	Cat.553772 RRID:AB_395041
Anti-Mouse CD45.1 Brilliant Violet 605 (2µl/10^6 cells)	Biolegend	Cat.110738 AB_10863973
Rat Anti-Mouse Gr-1 (Ly-6G and Ly-6C) PE (2µl/10^6 cells)	BD Biosciences	Cat.553128 RRID:AB_394644
Rat Anti-Mouse Mac-1 (CD11b) PE (2µl/10^6 cells)	BD Biosciences	Cat.557397 RRID:AB_396680
Rat Anti-Mouse CD3 V450 (2µl/10^6 cells)	BD Biosciences	Cat.561389 RRID:AB_10679120
Anti-Mouse CD41 Alexa Fluor 488 (2µl/10^6 cells)	Biolegend	Cat.133908 RRID:AB_10645332
Rat anti-Mouse CD150 Alexa Fluor 647 (2µl/10^6 cells)	BD Biosciences	Cat.562647 RRID:AB_2737701
Anti-Mouse CD34 Antibody PE (5µl/10^6 cells)	Biolegend	119308
Anti-Mouse CD34 Alexa Fluor® 647 (5µl/10^6 cells)	Biolegend	Cat: 119314
Rat anti-Mouse CD117 APC-H7 (2µl/10^6 cells)	BD Biosciences	Cat: 560185
Rat anti-Mouse c-kit (CD117) PE (2µl/10^6 cells)	BD Biosciences	Cat.553355
Anti-mouse Ly-6A/E (Sca-1) Brilliant Violet 605™ (2µl/10^6 cells)	Biolegend	Cat: 108133
Rat anti-Mouse Sca-1 (Ly-6A/E) APC-Cy7 (2µl/10^6 cells)	BD Biosciences	Cat.560654 RRID:AB_1727552
Lineage Antibody Cocktail V450 Mouse (20 µl/10^6 cells)	BD Biosciences	Cat.561301 RRID:AB_10611731
Anti-Mouse CD31-Alexa-647 (5µl/10^6 cells)	Biolegend	Cat. 102516 RRID:AB_2161029
Anti-Mouse CD31-Alexa-488 (2µl/10^6 cells)	Biolegend	Cat. 102414
Anti-CD31 Alexa-488 (5µl/10^6 cells)	R&D Systems	Cat. FAB3628G RRID:AB_10972784
Anti-Endomucin AF750 (2µl/10^6 cells)	Bioss Antibodies	Cat. bs-5884R-a750 RRID:AB_11090525

Annexin V-FITC (2µl/10^6 cells)	BD Biosciences	Cat: 556547
Anti-Mouse LGR5 Alexa-488 (2µl/10^6 cells)	R&D Systems	Cat: FAB8240G-100UG
Anti-LGR5 (5µl/10^6 cells)	Abcam	Cat: ab273092
FITC Mouse Anti-Ki-67 Set (2µl/10^6 cells)	BD Biosciences	Cat: 556026 **RRID:AB_396302**
Anti-Phospho-Cdk5 (Tyr15) (2µl/10^6 cells)	Origene	Cat: TA325347
Recombinant Anti-Semaphorin 3A antibody [EPR19367] (5µl/10^6 cells)	Abcam	Cat: ab199475
Recombinant Anti-Neuropilin 1 antibody [EPR3113] (5µl/10^6 cells)	Abcam	Cat: ab81321
Goat anti-Rabbit IgG (H+L) Cross-Adsorbed Secondary Antibody, Alexa Fluor 555 (1µl/10^6 cells)	Thermofisher	A-21428
Goat anti-Rabbit IgG (H+L) Superclonal™ Secondary Antibody, Alexa Fluor 488 (1µl/10^6 cells)	ThermoFisher	Cat: A27034
Donkey anti-Goat IgG (H+L) Cross-Adsorbed Secondary Antibody, Alexa Fluor 488 (1µl/10^6 cells)	ThermoFisher	Cat: A-11055
CellEvent Caspase-3/7 Green Flow Assay Kit	ThermoFisher	Cat: C10740
Lycopersicon Esculentum (Tomato) Lectin (LEL, TL), DyLight® 594 DL-1177-1	Vector Laboratories	Cat: DL-1177-1
Sema3A Polyclonal Antibody, HRP Conjugated (2µl/10^6 cells)	Bioss Antibodies	Cat: bs-10468R-HRP
Mouse R-Spondin 2 Antibody (2µl/10^6 cells)	R&D Systems	Cat: MAB32661-100
Normal Goat IgG Control	R&D Systems	Cat: AB-108-C
Fixable Viability Stain 660 (5µl/10^6 cells)	BD Biosciences	Cat: 564405
7-AAD (5µl/10^6 cells)	BD Biosciences	Cat: 559925
DAPI (1:1000)	Biolegend	Cat: 422801
Chemicals, Peptides, and Recombinant Proteins		
Liberase™ TL Research Grade	Sigma	Cat. 5401020001
Roscovitine	Tocris	Cat: 1332
Recombinant Mouse Semaphorin 3A Fc Chimera Protein	R&D Systems	Cat: 5926-S3-025/CF
Recombinant Human Semaphorin 3A Fc Chimera Protein, CF	R&D Systems	Cat: 1250-S3-025
Recombinant Mouse Neuropilin-1 Protein, CF	R&D Systems	Cat: 5994-N1-050
Sino Biological Mouse Semaphorin 3A / SEMA3A Protein (His Tag)	Fisher Scientific	Cat: 50-162-2235
Recombinant Mouse Wnt-3a Protein	R&D Systems	Cat: 1324-WN-002
Recombinant Mouse R-Spondin 2 Protein	R&D Systems	Cat: 6946-RS-025/CF
EDTA	Sigma Aldrich	E5134-500g
PVP	Sigma Aldrich	P5288-100g
Sucrose	Sigma Aldrich	S7903-1KG
Fluoromount-G	SouthernBiotech	0100-01
4% Paraformaldehyde in PBS	Fisher Scientific	NC9245948
Recombinant Mouse Thrombopoietin Protein, CF	R&D Systems	488-TO/CF
Recombinant Mouse SCF Protein, CF	R&D Systems	455-MC/CF
Recombinant Mouse Flt-3, CF	R&D Systems	427-FL/CF
Gelatin from porcine skin	Sigma Aldrich	G1890-100G
IMDM, Iscove's Modified Dulbecco's Medium	Life Technologies	12440061
Hyclone Fetal Bovine Serum	GE Healthcare	16777-014
Penicillin-Streptomycin	Gibco	15140148
Critical Commercial Assays		
Methocult GF M3434	STEMCELL Technologies	Cat. 03434
Miltenyi Lineage Cell Depletion kit	Miltenyi Biotec	Cat. 130-090-858

Taqman Gene Expression Master Mix	ThermoFisher	Cat. 4304437
High Capacity cDNA reverse transcription kit	ThermoFisher	Cat. 4368813
Mouse Sema3A ELISA	Biomatik	Cat. EKU07258
Mouse RSPO2 ELISA	Lifespan Bio	Cat: LS-F39113
LS Columns	Miltenyi Biotec	130-042-401
Experimental Models: Organisms/Strains		
Mouse: C57BL/6	Jackson Laboratories	JAX: 000664 **RRID:IMSR_JAX:000664**
Mouse: B6.SJL	Jackson Laboratories	JAX: 002014 **RRID:IMSR_JAX:002014**
Mouse: Sema3A FL/FL	RIKEN Repository	IMSR: RBRC01106 **RRID:IMSR_RBRC01106**
Mouse: Nrp1 FL/FL	Jackson Laboratories	JAX: 005247 **RRID:IMSR_JAX:005247**
Mouse: Cdh5-Cre	Taconic Laboratories	Taconic: 13073 **RRID:IMSR_TAC:13073**
Mouse: NRP1(VEGF-)	Dr. Chengua Gu	N/A
Mouse: p53(-/-)	Jackson Laboratories	JAX: 002101 **RRID:IMSR_JAX:002101**
Oligonucleotides		
Mouse GAPDH Taqman gene expression assay	ThermoFisher	Mm99999915_g1
Mouse Cdh5 Taqman gene expression assay	ThermoFisher	Mm00486938_m1
Mouse Sema3A Taqman gene expression assay	ThermoFisher	Mm00436469_m1
Mouse Nrp1 Taqman gene expression assay	ThermoFisher	Mm00435379_m1
Mouse Nrp2 Taqman gene expression assay	ThermoFisher	Mm00803099_m1
Mouse Sema6A Taqman gene expression assay	ThermoFisher	Mm00444441_m1
Mouse Sema7A Taqman gene expression assay	ThermoFisher	Mm00441361_m1
Mouse Sema3F Taqman gene expression assay	ThermoFisher	Mm00441325_m1
Mouse Lgr5 Taqman gene expression assay	ThermoFisher	Mm00438890_m1
Mouse Fzd8 Taqman gene expression assay	ThermoFisher	Mm00433419_s1
Mouse Lrp6 Taqman gene expression assay	ThermoFisher	Mm00999795_m1
Mouse Lgr4 Taqman gene expression assay	ThermoFisher	Mm00554385_m1
Mouse Lgr6 Taqman gene expression assay	ThermoFisher	Mm05916284_s1
Mouse Puma Taqman gene expression assay	ThermoFisher	Mm00519268_m1
Mouse Bax Taqman gene expression assay	ThermoFisher	Mm00432051_m1
Mouse Bak1 Taqman gene expression assay	ThermoFisher	Mm00432045_m1
Mouse Bad Taqman gene expression assay	ThermoFisher	Mm00432042_m1
Software and Algorithms		
FlowJo (v10)	TreeStar	www.flowjo.com
GraphPad Prism 6.0	GraphPad Software	www.graphpad.com
Fiji (Version 2.3.0/1.53f)	Image J	https://imagej.nih.gov/ij/
Pioneer QDAT software (Vers 3.41)	ForteBio	https://www.fortebio.com/sites/default/files/en/assets/brochures/pioneer-and-pioneer-fe-systems.pdf
Leica LAS X Version 5.0.2	Leica	https://www.leica-microsystems.com/products/microscope-software/p/leica-las-x-ls/

Zen 2 (blue edition)	Zeiss	https://www.zeiss.com/content/dam/Microscopy/Downloads/Pdf/FAQs/zen2-blue-edition_installation-guide.pdf
Tecan Spark Control Software InfiniteM1000 Pro	Tecan	
BD FACSDiva v 9.1	BD	https://www.bdbiosciences.com/en-eu/products/software/instrument-software/bd-facsdiva-software
