# Supplementary material for: Neuropilin 1 regulates bone marrow vascular regeneration and hematopoietic reconstitution
Source: Nat Commun. 2021 Nov 30;12:6990. doi: 10.1038/s41467-021-27263-y (PMC8635308; doi:10.1038/s41467-021-27263-y)
Supplement: Supplementary file 3 — Reporting Summary [file 41467_2021_27263_MOESM3_ESM.pdf]

Corresponding author(s): John P. Chute
Last updated by author(s): Oct 24, 2021

Reporting Summary

Nature Research wishes to improve the reproducibility of the work that we publish. This form provides structure for consistency and transparency in reporting. For further information on Nature Research policies, see our Editorial Policies and the Editorial Policy Checklist.

Statistics

For all statistical analyses, confirm that the following items are present in the figure legend, table legend, main text, or Methods section.

n/a	Confirmed	
☐	☒	The exact sample size (*n*) for each experimental group/condition, given as a discrete number and unit of measurement
☐	☒	A statement on whether measurements were taken from distinct samples or whether the same sample was measured repeatedly
☐	☒	The statistical test(s) used AND whether they are one- or two-sided<br>*Only common tests should be described solely by name; describe more complex techniques in the Methods section.*
☐	☒	A description of all covariates tested
☐	☒	A description of any assumptions or corrections, such as tests of normality and adjustment for multiple comparisons
☐	☒	A full description of the statistical parameters including central tendency (e.g. means) or other basic estimates (e.g. regression coefficient) AND variation (e.g. standard deviation) or associated estimates of uncertainty (e.g. confidence intervals)
☐	☒	For null hypothesis testing, the test statistic (e.g. *F*, *t*, *r*) with confidence intervals, effect sizes, degrees of freedom and *P* value noted<br>*Give P values as exact values whenever suitable.*
☒	☐	For Bayesian analysis, information on the choice of priors and Markov chain Monte Carlo settings
☒	☐	For hierarchical and complex designs, identification of the appropriate level for tests and full reporting of outcomes
☒	☐	Estimates of effect sizes (e.g. Cohen's *d*, Pearson's *r*), indicating how they were calculated

Our web collection on statistics for biologists contains articles on many of the points above.

Software and code

Policy information about availability of computer code

Data collection

Data collection:
- For flow cytometric data collection, the BD FACSDiva Software (v9.1) software was used
- For confocal microscopy data collection, the Leica LAS X Version 5.0.2 was used
- For brightfield microscopy data collection, the Zen 2 (blue) edition software was used
- For qRT-PCR data collection: the QuantStudio Analysis Software (ver 1.7.1) was used
- For plate reader assays, Spark Control Software InfiniteM1000 Pro

Data analysis	• For RNA sequence analysis: The Partek flow [1] and Ingenuity Pathway Analysis (IPA) [2] were used for bioinformatics methods and data analysis. Reads per gene were quantified using STAR - 2.7.2a [3] and mm10 (Ensembl GRCm38.97).<br><br>[1] Partek® Flow® software, version 7.0 Copyright ©; 2019 Partek Inc., St. Louis, MO, USA.<br><br>[2] IPA: Causal analysis approaches in Ingenuity Pathway Analysis. Bioinformatics. 2014 Feb 15;30(4):523-30.<br><br>[3] STAR: ultrafast universal RNA-seq aligner, A Dobin, CA Davis, F Schlesinger, J Drenkow, C Zaleski, S Jha, P Batut, Bioinformatics 29 (1), 15-21Fast gapped-read alignment with Bowtie 2. Langmead B, Salzberg SL. Nat Methods. (2012)<br><br>• For flow cytometric analysis, FlowJo (version 10.7.1) was used<br>• For microscopy analysis, Fiji (version 2.1.0/1.53c) was used<br>• For statistical analysis, GraphPad Prism (version 9.1.0) was used<br>• For surface plasmon resonance, Pioneer QDAT software (Version 3.41) was used

For manuscripts utilizing custom algorithms or software that are central to the research but not yet described in published literature, software must be made available to editors and reviewers. We strongly encourage code deposition in a community repository (e.g. GitHub). See the Nature Research guidelines for submitting code & software for further information.

Data

Policy information about availability of data

All manuscripts must include a data availability statement. This statement should provide the following information, where applicable:

- Accession codes, unique identifiers, or web links for publicly available datasets
- A list of figures that have associated raw data
- A description of any restrictions on data availability

The raw data files of RNA sequencing data have been deposited and are openly availability at the Gene Expression Omnibus GEO data repository under accession code (GSE149776, https://www.ncbi.nlm.nih.gov/geo/query/acc.cgi?acc=GSE149776). The MetazSecKB database is available at http://proteomics.ysu.edu/secretomes/animal/. All other data supporting the findings of this study are openly available within the Source Data File included in this manuscript.

Field-specific reporting

Please select the one below that is the best fit for your research. If you are not sure, read the appropriate sections before making your selection.

☒ Life sciences ☐ Behavioural & social sciences ☐ Ecological, evolutionary & environmental sciences

For a reference copy of the document with all sections, see nature.com/documents/nr-reporting-summary-flat.pdf

Life sciences study design

All studies must disclose on these points even when the disclosure is negative.

Sample size	For all animal survival studies a sample size of n =12 mice was used to achieve a 95% power to detect a mean difference of 1 standard deviation with a significance level of 0.05 using a Log-rank (Mantel-Cox) test. Competitive repopulation assays were performed with a sample size of n=10 mice and hematopoietic recovery studies were performed with n = 8 - 9 to achieve a 95% power to detect a mean difference of 1 standard deviation with a significance level of 0.05 using a two-tailed student t-test. Sample sizes were also contributed to by independent replicate experiments.
Data exclusions	No data were excluded from the analysis.
Replication	All attempts at replication were successful. All experiments were repeated in multiple independent experiments. Within the figure legends, the number of replicates is defined.
Randomization	All mice used in this study were weight, sex and age matched prior to their allocation into treatment groups.
Blinding	No blinding was performed because the data acquired were obtained by using objective quantitative methods and researchers did not have expectations regarding the outcome.

Reporting for specific materials, systems and methods

We require information from authors about some types of materials, experimental systems and methods used in many studies. Here, indicate whether each material, system or method listed is relevant to your study. If you are not sure if a list item applies to your research, read the appropriate section before selecting a response.

Materials & experimental systems

n/a	Involved in the study
	x Antibodies
x	Eukaryotic cell lines
x	Palaeontology and archaeology
	x Animals and other organisms
x	Human research participants
x	Clinical data
x	Dual use research of concern

Methods

n/a	Involved in the study
x	ChIP-seq
	x Flow cytometry
x	MRI-based neuroimaging

Antibodies

Antibodies used

Antibodies
Goat anti-Nrp1 (1:200)
Validation criteria: https://www.rndsystems.com/products/mouse-rat-neuropilin-1-antibody_af566 R&D Systems Cat. AF566;
RRID:AB_355445

Anti-mouse VE-Cadherin Alexa Fluor® 647 (1:200)
Validation criteria: https://www.biolegend.com/en-us/products/alexa-fluor-647-anti-mouse-cd144-ve-cadherin-antibody-6569
Biolegend Cat. 138006
RRID:AB_10569114

Anti-Leptin receptor PE (1:200)
Validation criteria: https://www.biossusa.com/products/bs-0961r-pe Bioss Antibodies Cat. bs-0961R-PE
RRID:AB_11051477

Rat Anti-Mouse B220 (CD45R) APC-Cy7 (2µl/106 cells)
Validation criteria: https://www.bdbiosciences.com/en-eu/products/reagents/flow-cytometry-reagents/research-reagents/single-color-antibodies-ruo/apc-cy-7-rat-anti-mouse-cd45r.552094 BD Biosciences Cat.552094
RRID:AB_394335

Rat Anti-Mouse CD45 V450 (2µl/106 cells)
Validation criteria: https://www.bdbiosciences.com/en-eu/products/reagents/flow-cytometry-reagents/research-reagents/single-color-antibodies-ruo/v450-rat-anti-mouse-cd45.560501
BD Biosciences Cat.560501; RRID:AB_1645275

Rabbit Anti-P53 (2µl/106 cells)
Validation criteria: https://www.cellsignal.com/products/primary-antibodies/p53-7f5-rabbit-mab/2527?site-search-type=Products&N=4294956287&Ntt=2527s&fromPage=plp&_requestid=2757103
Cell Signaling Cat: 2527S
RRID:AB_10695803

Mouse Anti-Phospho P53 (S15) (2µl/106 cells)
Validation criteria: https://www.cellsignal.com/products/primary-antibodies/phospho-p53-ser15-16g8-mouse-mab/9286?_=1635037496378&Ntt=9286S&tahead=true
Cell Signaling Cat: 9286S
RRID:AB_331741

Mouse Anti-CD45.2 FITC Mouse anti-Mouse (2µl/106 cells)
Validation criteria: https://www.bdbiosciences.com/en-eu/products/reagents/flow-cytometry-reagents/research-reagents/single-color-antibodies-ruo/fitc-mouse-anti-mouse-cd45-2.553772
BD Biosciences Cat.553772
RRID:AB_395041

Anti-Mouse CD45.1 Brilliant Violet 605 (2µl/106 cells)
Validation criteria: https://www.biolegend.com/en-us/products/brilliant-violet-605-anti-mouse-cd45-1-antibody-7850
Biolegend Cat.110738
AB_10863973

Rat Anti-Mouse Gr-1 (Ly-6G and Ly-6C) PE (2µl/106 cells)
Validation criteria: https://www.bdbiosciences.com/content/dam/bdb/products/global/reagents/flow-cytometry-reagents/research-reagents/single-color-antibodies-ruo/553128_base/pdf/553128.pdf
BD Biosciences Cat.553128
RRID:AB_394644

Rat Anti-Mouse Mac-1 (CD11b) PE (2µl/106 cells)
Validation criteria: https://www.bdbiosciences.com/en-eu/products/reagents/flow-cytometry-reagents/research-reagents/single-color-antibodies-ruo/pe-rat-anti-cd11b.557397
BD Biosciences Cat.557397
RRID:AB_396680

Rat Anti-Mouse CD3 V450 (2µl/106 cells)
Validation criteria: https://www.bdbiosciences.com/en-eu/products/reagents/flow-cytometry-reagents/research-reagents/single-color-antibodies-ruo/v450-rat-anti-mouse-cd3-molecular-complex.561389
BD Biosciences Cat.561389
RRID:AB_10679120

Anti-Mouse CD41 Alexa Fluor 488 (2µl/106 cells)
Validation criteria: https://www.biolegend.com/en-us/products/alexa-fluor-488-anti-mouse-cd41-antibody-6907
Biolegend Cat.133908
RRID:AB_10645332

Rat anti-Mouse CD150 Alexa Fluor 647 (2µl/106 cells)
Validation criteria: https://www.bdbiosciences.com/en-us/products/reagents/flow-cytometry-reagents/research-reagents/single-color-antibodies-ruo/alexa-fluor-647-rat-anti-mouse-cd150.562647
BD Biosciences Cat.562647
RRID:AB_2737701

Anti-Mouse CD34 Antibody PE (5µl/106 cells)
Validation criteria: https://www.biolegend.com/en-us/products/pe-anti-mouse-cd34-antibody-3072
Biolegend 119308

Anti-Mouse CD34 Alexa Fluor® 647 (5µl/106 cells)
Validation criteria: https://www.biolegend.com/en-us/products/alexa-fluor-647-anti-mouse-cd34-antibody-3773
Biolegend Cat: 119314

Rat anti-Mouse CD117 APC-H7 (2µl/106 cells)
Validation criteria: https://www.bdbiosciences.com/en-us/products/reagents/flow-cytometry-reagents/research-reagents/single-color-antibodies-ruo/apc-h7-rat-anti-mouse-cd117.560185

BD Biosciences Cat: 560185
Rat anti-Mouse c-kit (CD117) PE (2µl/106 cells)
Validation criteria: https://www.bdbiosciences.com/en-us/products/reagents/flow-cytometry-reagents/research-reagents/single-color-antibodies-ruo/pe-rat-anti-mouse-cd117.553355

BD Biosciences Cat.553355
Anti-mouse Ly-6A/E (Sca-1) Brilliant Violet 605™ (2µl/106 cells)
Validation criteria: https://www.biolegend.com/en-us/products/brilliant-violet-605-anti-mouse-ly-6a-e-sca-1-antibody-8664
Biolegend Cat: 108133

Rat anti-Mouse Sca-1 (Ly-6A/E) APC-Cy7 (2µl/106 cells)
Validation criteria: https://www.bdbiosciences.com/en-us/products/reagents/flow-cytometry-reagents/research-reagents/single-color-antibodies-ruo/apc-cy-7-rat-anti-mouse-ly-6a-e.560654
BD Biosciences Cat.560654
RRID:AB_1727552

Lineage Antibody Cocktail V450 Mouse (20 µl/106 cells)
Validation criteria: https://www.bdbiosciences.com/en-us/products/reagents/flow-cytometry-reagents/research-reagents/panels-multicolor-cocktails-ruo/v450-mouse-lineage-antibody-cocktail-with-isotype-control.561301
BD Biosciences Cat.561301
RRID:AB_10611731

Anti-Mouse CD31-Alexa-647 (5µl/106 cells)
Validation criteria: https://www.biolegend.com/en-us/products/alexa-fluor-647-anti-mouse-cd31-antibody-3094
Biolegend Cat. 102516
RRID:AB_2161029

Anti-Mouse CD31-Alexa-488 (2µl/106 cells)
Validation criteria: https://www.biolegend.com/en-us/products/alexa-fluor-488-anti-mouse-cd31-antibody-3091
Biolegend Cat. 102414

Anti-CD31 Alexa-488 (5µl/106 cells)
Validation criteria: https://www.rndsystems.com/products/mouse-rat-cd31-pecam-1-alexa-fluor-488-conjugated-antibody_fab3628g

R&D Systems Cat. FAB3628G
RRID:AB_10972784

Anti-Endomucin AF750 (2µl/106 cells)
Validation criteria: https://www.biossusa.com/products/bs-5884r-a750
Bioss Antibodies Cat. bs-5884R-a750
RRID:AB_11090525

Annexin V-FITC (2µl/106 cells)
Validation criteria: https://www.bdbiosciences.com/en-us/products/reagents/flow-cytometry-reagents/research-reagents/panels-multicolor-cocktails-ruo/fitc-annexin-v-apoptosis-detection-kit-i.556547
BD Biosciences Cat: 556547

Anti-Mouse LGR5 Alexa-488 (2µl/106 cells)
Validation criteria: https://www.rndsystems.com/products/mouse-lgr5-gpr49-alexa-fluor-488-conjugated-antibody-803420_fab8240g
R&D Systems Cat: FAB8240G-100UG

Anti-LGR5 (5µl/106 cells)
Validation criteria: https://www.abcam.com/lgr5-antibody-oti2a2-ab273092.html
Abcam Cat: ab273092

FITC Mouse Anti-Ki-67 Set (2µl/106 cells)
Validation criteria: https://www.bdbiosciences.com/en-us/products/reagents/flow-cytometry-reagents/research-reagents/panels-multicolor-cocktails-ruo/fitc-mouse-anti-ki-67-set.556026
BD Biosciences Cat: 556026
RRID:AB_396302

Anti-Phospho-Cdk5 (Tyr15) (2µl/106 cells)
Validation criteria: https://www.origene.cn/catalog/antibodies/primary-antibodies/ta325347/cdk5-rabbit-polyclonal-antibody
Origene Cat: TA325347

Recombinant Anti-Semaphorin 3A antibody [EPR19367] (5µl/106 cells)
Validation criteria: https://www.abcam.com/semaphorin-3a-antibody-epr19367-ab199475.html
Abcam Cat: ab199475

Recombinant Anti-Neuropilin 1 antibody [EPR3113] (5µl/106 cells)
Validation criteria: https://www.abcam.com/neuropilin-1-antibody-epr3113-ab81321.html
Abcam Cat: ab81321

Goat anti-Rabbit IgG (H+L) Cross-Adsorbed Secondary Antibody, Alexa Fluor 555 (1µl/106 cells)
Validation criteria: https://www.thermofisher.com/antibody/product/Goat-anti-Rabbit-IgG-H-L-Cross-Adsorbed-Secondary-Antibody-Polyclonal/A-21428
Thermofisher A-21428

Goat anti-Rabbit IgG (H+L) Superclonal™ Secondary Antibody, Alexa Fluor 488 (1µl/106 cells)
Validation criteria: https://www.thermofisher.com/antibody/product/Goat-anti-Rabbit-IgG-H-L-Secondary-Antibody-Recombinant-Polyclonal/A27034
ThermoFisher Cat: A27034

Donkey anti-Goat IgG (H+L) Cross-Adsorbed Secondary Antibody, Alexa Fluor 488 (1µl/106 cells)
Validation criteria: https://www.thermofisher.com/antibody/product/Donkey-anti-Goat-IgG-H-L-Cross-Adsorbed-Secondary-Antibody-Polyclonal/A-11055
ThermoFisher Cat: A-11055

Sema3A Polyclonal Antibody, HRP Conjugated (2µl/106 cells)
Validation criteria: https://www.biossusa.com/products/bs-10468r-hrp
Bioss Antibodies Cat: bs-10468R-HRP

Mouse R-Spondin 2 Antibody (2µl/106 cells)
Validation criteria: https://www.rndsystems.com/products/mouse-r-spondin-2-antibody-773029_mab32661 R&D Systems Cat: MAB32661-100

Normal Goat IgG Control
Validation criteria: https://www.rndsystems.com/products/normal-goat-igg-control_ab-108-c
R&D Systems Cat: AB-108-C

Validation

All antibodies used are validated for detection of indicated proteins and specificity was confirmed by comparison to isotype control

staining. All antibodies used in this study were commercially available by vendors and have been validated by the manufacturers. Validation and specific validation criteria can be found at the following vendor websites above.

Animals and other organisms

Policy information about studies involving animals; ARRIVE guidelines recommended for reporting animal research

Laboratory animals

In this study we used male and female mice that were 8-12 weeks of age of the following strains:

Mouse: C57BL/6 Jackson Laboratories JAX: 000664
RRID:IMSR_JAX:000664
Mouse: B6.SJL Jackson Laboratories JAX: 002014
RRID:IMSR_JAX:002014
Mouse: Sema3A FL/FL RIKEN Repository IMSR: RBRC01106
RRID:IMSR_RBRC01106
Mouse: Nrp1 FL/FL Jackson Laboratories JAX: 005247
RRID:IMSR_JAX:005247
Mouse: Cdh5-Cre Taconic
Laboratories Taconic: 13073
RRID:IMSR_TAC:13073
Mouse: NRP1(VEGF-) Dr. Chengua Gu N/A
Mouse: p53(-/-) Jackson
Laboratories JAX: 002101
RRID:IMSR_JAX:002101

Animals were housed in a clean barrier facility with room temperature conditions (20-26 °C), relative humidity 30-70%, with 12-hour dark/light cycles.

Wild animals

No wild animals were used in this study .

Field-collected samples

No field-collected samples were used in this study.

Ethics oversight

All animal procedures were performed in accordance with UCLA Animal Research Committee-approved protocol (ARC #2014-021-13P, principal investigator: JPC) and Cedars Sinai Medical Center Animal Use Committee-approved protocol (IACUC009617).

Note that full information on the approval of the study protocol must also be provided in the manuscript.

Flow Cytometry

Plots

Confirm that:

- [x] The axis labels state the marker and fluorochrome used (e.g. CD4-FITC).
- [x] The axis scales are clearly visible. Include numbers along axes only for bottom left plot of group (a 'group' is an analysis of identical markers).
- [x] All plots are contour plots with outliers or pseudocolor plots.
- [x] A numerical value for number of cells or percentage (with statistics) is provided.

Methodology

Sample preparation	Detailed sample preparation and source of samples are listed in Methods.
Instrument	BD FACS Canto II and FACS Aria II
Software	BD FACSDIVA (BD Biosciences, NJ) for collection and FlowJo v9 (Treestar, Ashland, OR) for analysis.
Cell population abundance	10,000 cells were sorted and purity was assessed by expression of endothelial surface markers.
Gating strategy	Relevant gating strategies shown in corresponding flow plots and Supplementary Information. Positive gates were set to levels where isotype staining was <1%.

- [x] Tick this box to confirm that a figure exemplifying the gating strategy is provided in the Supplementary Information.
